# Streptococcus mutans and Actinomyces naeslundii Interaction in Dual-Species Biofilm

**DOI:** 10.3390/microorganisms8020194

**Published:** 2020-01-31

**Authors:** Rosa Virginia Dutra de Oliveira, Fernanda Salloume Sampaio Bonafé, Denise Madalena Palomari Spolidorio, Cristiane Yumi Koga-Ito, Aline Leite de Farias, Kelly R. Kirker, Garth A. James, Fernanda Lourenção Brighenti

**Affiliations:** 1 School of Dentistry, São Paulo State University (UNESP), Araraquara, SP 14801-385, Brazil; drarosadutra@hotmail.com (R.V.D.d.O.); fernandassbonaf@foar.unesp.br (F.S.S.B.); denise.mp.spolidorio@unesp.br (D.M.P.S.); aline.leitefarias@gmail.com (A.L.d.F.); 2 São José dos Campos Institute of Science and Technology, São Paulo State University (UNESP), São José dos Campos, SP 12245-000, Brazil; cristiane.koga-ito@unesp.br; 3 Centre for Biofilm Engineering, Montana State University, Bozeman, MT 59717, USA; kelly.kirker@montana.edu (K.R.K.); gjames@montana.edu (G.A.J.)

**Keywords:** microbial interaction, biofilm, virulence factor, dental caries, *S. mutans*, *A. naeslundii*

## Abstract

The study of bacterial interaction between *Streptococcus mutans* and *Actinomyces naeslundii* may disclose important features of biofilm interspecies relationships. The aim of this study was to characterize—with an emphasis on biofilm formation and composition and metabolic activity—single- and dual-species biofilms of *S. mutans* or *A. naeslundii,* and to use a drip flow reactor (DFR) to evaluate biofilm stress responses to 0.2% chlorhexidine diacetate (CHX). Single- and dual-species biofilms were grown for 24 h. The following factors were evaluated: cell viability, biomass and total proteins in the extracellular matrix, 2,3-bis(2-methoxy-4-nitro-5-sulfophenyl)-2H-tetrazolium-5-carboxanilide—“XTT”—reduction and lactic acid production. To evaluate stress response, biofilms were grown in DFR. Biofilms were treated with CHX or 0.9% sodium chloride (NaCl; control). Biofilms were plated for viability assessment. Confocal laser-scanning microscopy (CLSM) was also performed. Data analysis was carried out at 5% significance level. *S. mutans* viability and lactic acid production in dual-species biofilms were significantly reduced. *S. mutans* showed a higher resistance to CHX in dual-species biofilms. Total protein content, biomass and XTT reduction showed no significant differences between single- and dual-species biofilms. CLSM images showed the formation of large clusters in dual-species biofilms. In conclusion, dual-species biofilms reduced *S. mutans* viability and lactic acid production and increased *S. mutans’* resistance to chlorhexidine.

## 1. Introduction

Improving the knowledge of the ecological composition of dental biofilm has promoted a major change in the control of biofilm-associated diseases [1,2,3]. Dental biofilm is a highly organized and structured community in which microbial cells are embedded in an extracellular matrix [4]. Kistler et al., 2015 [5] estimated that there are 250 species-level operational taxonomic units (OTUs) in dental biofilm. Physical, metabolic and molecular interactions between these species determines how biofilm will attach, grow and survive [6]. One of the most well-known outcomes of this bacterial interaction is an increased resistance to antimicrobial substances in comparison to cells in suspension [7,8].

The ecological relationship within dental biofilm is complex and comprises a series of mechanisms, which can either benefit or harm bacterial species. Examples of beneficial interactions include: (a) cellular signalling and communication during low pH cycles, which are able to promote a shift in the virulence of non-mutans streptococci and *Actinomyces* species, increasing the acid production of biofilms [9]; (b) oxygen consumption by *Neisseria* spp. Which enables the growth of anaerobic species in the presence of oxygen [10]; (c) protection against oxidative stress observed, for instance, in the coaggregation between *Actinomyces naeslundii* and *Streptotoccus gordonii* [11]; (d) the production of an extracellular polyssacharides matrix, which enables the adhesion of other microbial species [12]. (e) the aggregation of other bacterial species, such as the one promoted by *Fusobacterium nucleatum*, a bridge bacterium between aerobic and anaerobic species [13]. A good example of competition within biofilms is the antagonistic relationship between *S. mutans* and *Streptococcus sanguinis*. *S. sanguinis* produces hydrogen peroxide, which inhibits *S. mutans* growth. On the other hand, *S. mutans* produces bacteriocins that inhibit *S. sanguinis* growth [14]. *S. sanguinis* growth may also be inhibited by the production of organic acids [15].

Despite the known importance of microbial interaction in dental biofilms, there are still many questions to be explored. Most of the studies in the literature have focused on understanding the gene expression of bacterial interaction [12,16,17,18]. However, gene expression studies are limited because they do not provide information on the biofilm phenotypic characteristics and virulence [19]. Thus, little is known about the phenotypic characteristics of biofilms when cariogenic species are combined.

Substantial efforts have been dedicated to understanding *S. mutans* virulence factors [20,21,22,23] because this species is strongly associated with dental caries [24]. However, with the deeper understanding of microbial physiology within biofilms, the importance of bacterial interaction for dental biofilm cariogenicity has been recognized. Studies have shown that the association of *S. mutans* with other microbial species leads to important changes in biofilm cariogenicity. The association with *Veilonella parvula* increased *S. mutans’* resistance to chlorhexidine, changed biofilm spatial arrangement and changed *S. mutans* gene expression [25,26,27]. The association with bifidobacteria promoted higher acid production [28]. The association with *Candida albicans* increased the concentration of water insoluble extracellular polysaccharides and led to the formation of a more complex biofilm with increased resistance to environmental stresses [29]. 

*A. naeslundii* has been correlated with biofilm ecological balance and is frequently associated with a healthy microbiota [9,10,11,12,13,14,15,16,17,18,19,20,21,22,23,24,25,26,27,28,29,30]. This species can increase local pH by producing ammonia and alkali [31,32] and can also metabolize lactic acid into weaker acids [33]. However, when biofilms are frequently exposed to acid challenges, this species can express a more pathogenic phenotype and further contribute to the development of dental caries [9,30]. To date, the role of this species in caries initiation is not well understood [19,24]. 

The study of bacterial interaction between *S. mutans* and *A. naeslundii* may disclose important features of biofilm interspecies relationships, such as their influence on biofilm metabolism and antimicrobial resistance. Therefore, the present study aimed to first characterize single- and dual-species biofilms of *S. mutans* and *A. naeslundii* by biofilm formation and composition (cell viability, biomass, and total protein concentration in the extracellular matrix) and metabolic activity (2,3-bis(2-methoxy-4-nitro-5-sulfophenyl)-2H-tetrazolium-5-carboxanilide—“XTT”—reduction and lactic acid production). Cell viability is an important indicator of biofilm cariogenicity, since higher abundance of cariogenic species—particularly *S. mutans*—increases virulence [9]. Biomass by crystal violet staining is based on the staining of live and dead cells and the extracellular matrix, which provides the overall information of the total attached biofilm. This has been used in different studies due to its high reproducibility and fast analysis and is a good indicator of caries activity [34,35]. The presence of specific proteins in the extracellular matrix has recently been shown to display an important role in the pathogenicity of dental biofilm [36,37,38]. XTT reduction provides information about overall bacterial metabolic activity, including cellular respiration, growth and reproduction [9,31,39]. Lactic acid is a crucial factor in the development of carious lesions, since its production is related to the demineralization of dental surface [9]. Furthermore, a biofilm reactor was used to evaluate the stress response of single- and dual-species biofilms after exposure to 0.2% chlorhexidine diacetate.

## 2. Materials and Methods

### 2.1. Characterization of Single- and Dual-Species Biofilms

Frozen stocks of *Streptococcus mutans* UA159 and *Actinomyces naeslundii* ATCC 12104 were stored in Brain Heart Infusion Broth (BHI broth, Himedia, Mumbai, India), supplemented with 20% glycerol, at −20 °C. The reference strains were grown in Brain Heart Infusion agar (BHI agar, Himedia, Mumbai, India) for *S. mutans,* and BHI agar supplemented with 10% defibrinated sheep blood for *A. naeslundii*. The plates were incubated in 5% CO_2_ at 37 °C for 72 h.

Biofilms were formed on sterilized hydroxyapatite disks [40] using an active adherence model (based on Exterkate et al. [41] and Albuquerque et al. [42]). Three culture conditions were used: (1) *S. mutans* single-species biofilms, (2) *A. naeslundii* single-species biofilms and (3) dual-species biofilms of *S. mutans* and *A. naeslundii*. 

Standardized bacterial suspensions in sterilized 0.9% NaCl containing 1 × 10^8^ CFU·mL^−1^ of *A. naeslundii* or 1 × 10^4^ CFU·mL^−1^ of *S. mutans* were prepared. The appropriate concentration of each bacterial species for growing dual-species biofilms was determined by pilot studies (Supplementary Material 1), using the values described by Xiao et al. as a starting point [43]. For the growth of single-species biofilms, 3 mL of each bacterial suspension was individually added to 27 mL of BHI broth supplemented with 0.2% sucrose (*v*/*v*) (BHI-S broth). For the growth of dual-species biofilms, 3 mL of *S. mutans* suspension was combined with 3 mL of *A. naeslundii* suspension in 24 mL of BHI-S broth. Biofilms grew on the surface of hydroxyapatite discs (*n* = 6) in BHI-S broth (1.5 mL) for 24 h at 37 °C/5% CO_2_. The experiment was repeated on three different occasions.

After 24 h of incubation, biofilms were characterized by the following factors: cell viability, biomass, and total protein concentration in the extracellular matrix and metabolic activity (lactic acid production and 2,3-bis(2-methoxy-4-nitro-5-sulfophenyl)-2H-tetrazolium-5-carboxanilide—“XTT”- reduction). A total of five sets of biofilms were grown, one set for each analysis carried out. All experiments were repeated on three independent occasions, using six discs per group. 

For viable cell quantification, the discs were washed by immersion ten times in 0.9% NaCl and biofilms were dispersed in 2 mL 0.9% NaCl by sonication (10 s, 42 kHz) [42]. Single- and dual-species biofilm suspensions were plated in triplicate in BHI blood agar. The plates were incubated in 5% CO_2_ at 37 °C for 72 h. Differential counting was carried out as previously standardized in pilot studies (Supplementary Material 1). Bacterial viability was expressed as the mean ± standard deviation log CFU·mL^−1^.

For total protein quantification in the extracellular matrix, biofilms were washed and re-suspended as previously described. The resulted suspension was vortexed and centrifuged (3000× *g*, 10 min at 4 °C; centrifuge model 5804R, Eppendorf, Hamburg, Germany). The supernatants were filtered using cellulose acetate membrane (pore size 0.2 µm; Chromafil Xtra CA-20/25, Macherey-Nagel, Durem, Germany) [44] and frozen at −20 °C until analysis. Protein quantification was carried out for the supernatant using the BCA kit (Thermo Scientific, Rockford, IL, USA), according to the manufacturer’s instructions. The absorbance was read at 562 nm and the values were converted to µg·mL^−1^, using the results obtained from a calibration curve made with albumin. Total protein was expressed as µg·mL^−1^.

Biofilm biomass was evaluated using crystal violet staining [45]. Biofilms were washed as previously described. Next, biofilms were fixed with 99.5% ethanol for 15 min. After drying at room temperature for 20 min, hydroxyapatite discs were immersed in 1% crystal violet solution. After 5 min, the discs were washed and dried at room temperature as previously described [45]. Biofilms were then immersed in 33% acetic acid. A volume of 200 μL of each well was transferred in triplicate to 96-well plates and the absorbance was read at 570 nm. Biofilm biomass was expressed as absorbance. 

For lactic acid production evaluation, after growth for 24 h, the culture medium was refreshed and the biofilms were incubated for an additional 3.5 h at 5% CO_2_ and 37 °C. Next, lactic acid concentration was determined in the culture medium [46] using the enzymatic method [47]. Samples were diluted at 1:4 *v*/*v* (culture medium: MiliQ water). The absorbance was read at 340 nm and the values were converted to mM, according to the readings from a calibration curve made with lactic acid. Lactic acid production was expressed as mM. 

XTT reduction was evaluated according to the methodology described by Cheng et al. [48]. Biofilms were washed as previously described. Hydroxyapatite discs were immersed in XTT-menadione solution (1 mg XTT/mL PBS; 0.4 mM menadione; Sigma Aldrich, St. Louis, MO, USA; 1.5 mL/well). Twenty-four-well plates were incubated for 3 h at 37 °C. Next, 200 μL of the XTT-menadione solution was transferred in triplicate to 96-well plates and the absorbance was read at 490 nm. XTT reduction was expressed as absorbance.

### 2.2. Stress Response of Single and Dual-Species Biofilms Using Drip-Flow Reactor

*S. mutans* ATCC 25175 and *A. naeslundii* ATCC 12104 were stored and grown under the same conditions as described previously, except that the substrate used was hydroxyapatite-coated glass slides. Biofilms were grown in drip flow reactor (DFR, BioSurface Technologies Inc., Bozeman, MT USA). The DFR consists of a polysulfone reactor body containing six parallel channels. Each channel fits one hydroxyapatite-coated glass slide (Clarkson Chromatography Products Inc., South Williamsport, PA, USA). Thus, it is possible to run six samples per experiment. Before starting the experiments, the DFR- and hydroxyapatite-coated glass slides inside were autoclaved for 15 min at 121 °C. The use of DFR was validated in pilot studies (Supplementary Material 2).

The same inoculum densities used to grow static biofilms (biofilm characterization assay) were used to grow *S. mutans* and *A. naeslundii* for DFR assays. Three culture conditions were used: (1) *S. mutans* single-species biofilms, (2) *A. naeslundii* single-species biofilms and (3) dual-species biofilms of *S. mutans* and *A. naeslundii*. The following treatment solutions were applied: (1) 0.2% chlorhexidine (diacetate salt, MP Biomedicals, Solon, OH, USA); (2) negative control, sterile saline solution (0.9% NaCl). 

DFR channels were inoculated with 1.0 mL of cell suspension (single-species biofilms) or with 500 μL of each bacterial inoculum (dual-species biofilms). DFR was incubated at a horizontal position for 1 h at 37 °C/5% CO_2_. This static incubation was to ensure bacterial attachment to the slides before the flow was initiated. Next, the reactors were inclined at 10° and medium flow was initiated at a 10 mL/h rate in each channel. This flow was used because it is approximately the normal non-stimulated salivary flow rate [49]. The medium consisted of full-strength BHI broth (Difco, Sparks, MD, USA) supplemented with 0.5% sucrose. 

After 24 h, each channel was rinsed with 10 mL of 0.9% NaCl to remove residual growth medium and planktonic cells. DFR was placed in a horizontal position and biofilms were treated for 2 min with 20 mL of 0.9% NaCl (control) or 0.2% chlorhexidine solution, freshly prepared on the day of use (diacetate salt; MP Biomedicals, Solon, OH, USA). After treatments, each DFR channel was rinsed with sterile saline solution (NaCl 0.9%) at a flow rate of 10 mL/h, in order to simulate salivary clearance [50].

Next, the slides were removed from the DFR and scraped thoroughly with a Teflon scraper into 10 mL of 0.9% NaCl. Slides and cell suspensions were vortexed for 30 s, sonicated for 2 min, and vortexed for an additional 30 s to remove and disperse the biofilm cells. Cell suspensions were serially diluted and plated on BHI agar supplemented with 10% sheep blood (Difco, Sparks, MD, USA). Plates were incubated for 48 h at 37 °C/5% CO_2_. The number of colony forming units was counted and the results were expressed as log CFU/mL. Experiments were repeated in five independent experiments, yielding a sample size of five glass slides for each type of biofilm and treatment solution.

Another batch of biofilms was cultivated under the same conditions as described above for confocal laser-scanning microscopy (CLSM) analyses. Biofilms were stained using Live/Dead BacLight Viability kit (Thermo Fisher Scientific, Eugene, OR, USA), which is comprised by SYTO9 and propidium iodide to differentiate bacterial cells without damage (fluorescent green) and bacterial cells with damaged membranes (fluorescent red). Images were examined using a Leica SP5 upright confocal laser-scanning microscope (Leica Microsystems Inc., Wetzlar, Germany) with a 63× water immersion objective at 1024 × 1024 pixels resolution. Biofilms’ sections were obtained at ten random positions on the glass slides. Image processing was performed using the Imaris Program (Bitplane Inc., Zurich, Switzerland).

### 2.3. Data Analysis

Initial biofilm characterization was analyzed by Software GraphPad Prism 3.02 (GraphPad Software Inc., San Diego, CA, USA). For bacterial viability (log CFU·mL^−1^), data showed normal distribution after the removal of one outlier from the *S. mutans* single-species group and one outlier from *A. naeslundii* dual-species group (*p* ≥ 0.3104). For lactic acid production and XTT reduction, data showed a normal distribution (*p* ≥ 0.0680 and *p*≥ 0.1451, respectively). For biomass and protein in extracellular matrix, data showed a non-normal distribution. Data that showed normal distribution (bacterial viability, lactic acid production and XTT reduction) were analyzed by one-way ANOVA, followed by Tukey’s test. Data from biomass and protein production were analyzed by Kruskal–Wallis test. The significance level was set at 5%.

For the stress response assay, data were analysed using IBM SPSS Statistics (SPSS, Chicago, IL, USA). The bacterial viability of DFR validation was analysed using unpaired *t*-test. For interspecies interaction evaluation, two factors were considered: (1) biofilm type (single- or dual-species biofilms) and (2) treatment solution (saline or chlorhexidine). Data showed equality of variances (Levene’s test) and normal distribution (Kolmogorov–Smirnov test). Two-way ANOVA followed by Tukey post hoc test was performed. The significance level was set at 5% for both studies. Confocal microscopy images were analysed descriptively. 

## 3. Results

### 3.1. Characterization of Single and Dual Species Biofilms

**Figure 1:**
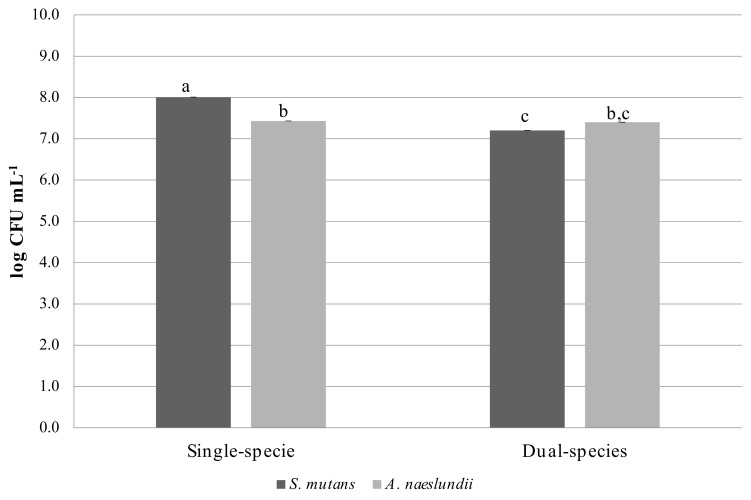
Characterization of single- and dual-species biofilms grown in a static model. Cell viability of *S. mutans* and *A. naeslundii*, according to the growth condition (log CFU·mL^−1^; mean ± standard deviation). Different letters show statistically significant differences (ANOVA, Tukey test; 5% significance). All experiments were repeated on three independent occasions, using six discs per group.

*A. naeslundii* viability was not affected by the presence of *S. mutans* in dual-species biofilms when compared to *A. naeslundii* single-species biofilms (Figure 1). *S. mutans* viability in dual-species biofilms was reduced by 10.11% in the presence of *A. naeslundii*. The percentage reduction of mean cell viability from *A. naeslundii* biofilms single-species biofilms to dual-species biofilms was 0.40%. 

**Figure 2:**
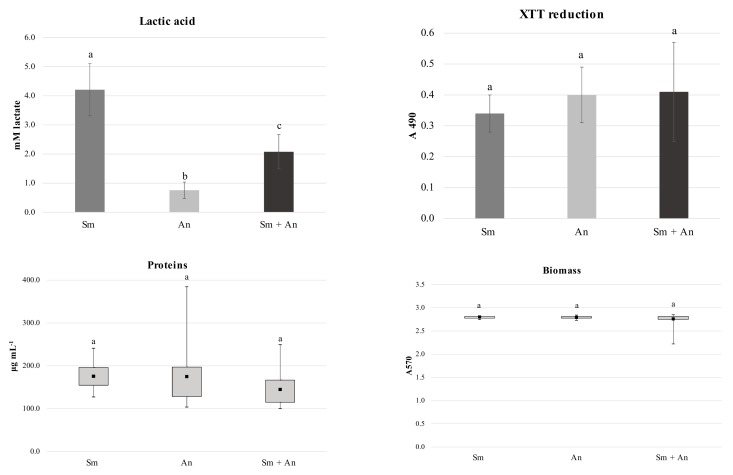
Characterization of single- and dual-species biofilms grown in a static model. lactic acid production, XTT reduction, and total protein concentration in biofilm matrix and biomass, according to the growth condition. Sm: *Streptococcus mutans* single-species biofilm; An: *Actinomyces naeslundii* single-species biofilm; Sm + An: *S. mutans* and *A. naeslundii* dual-species biofilm. Different letters show statistical significant differences (5% significance). Lactic acid and XTT reduction: ANOVA; mean ± standard error. Proteins and biomass: Kruskal–Wallis; median and quartiles. All experiments were repeated on three independent occasions, using six discs per group.

Lactic acid production in dual-species biofilms was reduced by 50% in comparison to *S. mutans* single-species biofilms. *S. mutans* single-species biofilms produced significantly more lactic acid in comparison to *A. naeslundii* single-species and dual-species biofilms. The amount of lactic acid produced by *A. naeslundii* single-species biofilms was the lowest observed in the present study (Figure 2; *p* < 0.0001). For the other cariogenicity factors analyzed (total protein concentration in biofilm matrix, biomass and XTT reduction) there were no statistically significant differences between the single- and dual-species biofilms (Figure 2; *p* = 0.6658; 0.9172 and 0.1549, respectively).

### 3.2. Stress Response of Single- and Dual-Species Biofilms Using Drip-Flow Reactor

Two-way ANOVA for bacterial viability after treatment with chlorhexidine showed a significant interaction between the two factors analysed (treatment solution and biofilm type; *p* = 0.001). Moreover, statistically significant differences were observed for the factor treatment solution (*p* < 0.001) but not for the factor culture condition (*p* = 0.261) (Supplementary Material 3).

**Figure 3:**
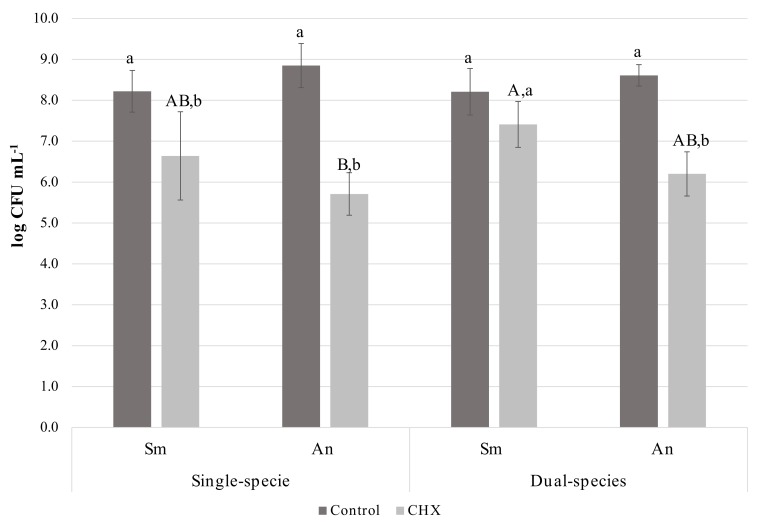
Stress response of single- and dual-species biofilms using DFR. Viability (mean ± sd; Log CFU/mL) in single- and dual-species biofilms of *S. mutans* and *A. naeslundii*. An single: *A. naeslundii* in single-species biofilms; An dual: *A. naeslundii* in dual-species biofilms; Sm single: *S. mutans* in single-species biofilms; Sm dual: *S. mutans* in dual-species biofilms. CHX: chlorhexidine diacetate. Means followed by different uppercase letters indicate statistically significant differences within chlorhexidine treatment (column). Means followed by different lowercase letters indicate statistically significant difference within each growth condition (rows) (two-way ANOVA and Tukey test, *p* < 0.05).

**Figure 4:**
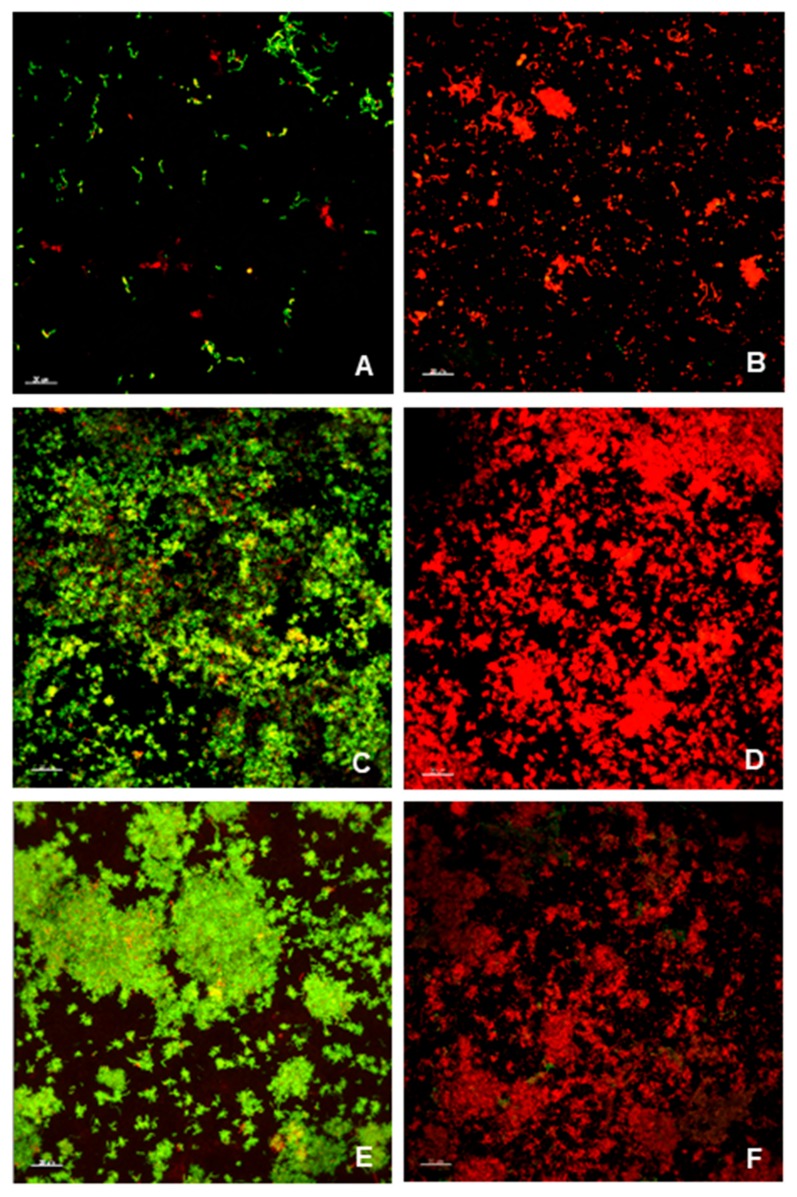
*S. mutans* and *A. naeslundii* biofilms—A: non-treated (NaCl) *S. mutans* biofilms; B: treated (CHX) *S. mutans* biofilms; C: non-treated (NaCl) *A. naeslundii* biofilms; D: treated (CHX) *A. naeslundii* biofilms; E: non-treated (NaCl) *S. mutans* and *A. naeslundii* biofilms; F: treated (CHX) *S. mutans* and *A. naeslundii* biofilms. Scale bar: 20 µm

Within chlorhexidine treated biofilms, *S. mutans* viability in dual-species biofilms showed a higher resistance to CHX when compared to *A. naeslundii* in single-species biofilm. Within the same culture condition, bacterial viability after chlorhexidine treatment was significantly reduced in all groups, except for *S. mutans* in dual-species biofilms (Figure 3). CLSM images showed that *S. mutans* was sparsely distributed in single-species biofilms, with no signs of clustering. In contrast, *A. naeslundii* single-species biofilms had cell aggregation. The formation of large clusters of *S. mutans* and *A. naeslundii* was observed in dual-species biofilms (Figure 4).

## 4. Discussion

Unraveling how microbial interactions take place in dental biofilm is an important step towards bringing new insights into caries’ development and establishing effective preventive and therapeutic approaches. In the present study, the interaction between *S. mutans* and *A. naeslundii* was characterized. The present study also implemented the use of a flow reactor to evaluate the stress response of single- and dual-species biofilms. 

The species studied included a key species for the maintenance of biofilm ecological balance that can also be present in incipient or radicular caries (*A. naeslundii*) and *Streptococcus mutans*, considered one of the most important cariogenic species [20,21,22,23,24]. The results of the present study showed that interactions between *S. mutans* and *A. naeslundii* occur during early stages of biofilm formation, leading to a decrease in lactic acid production and to a higher resistance of *S. mutans* to chlorhexidine exposure. Furthermore, the use of the active adherence model [41,42] allows biofilm formation only with the cells able to adhere to the substrate, which prevents microorganism deposition by the action of gravity. This is a way to reduce limitations related to static biofilm growth and to mimic the oral milieu.

The greatest number of viable cells of *S. mutans* in single-species biofilms may be related to the faster cell division of *S. mutans* in comparison to *A. naeslundii* [51]. Pilot studies carried out to standardize dual-species biofilm growth showed that *S. mutans* reaches exponential phase growth 3 h after initial inoculation, while *A. naeslundii* reached this phase after 22.5 h of initial inoculation (data not shown). This difference in growth rate was considered during the establishment of dual-species biofilms. Moreover, the number of viable bacteria in single-species biofilms were similar to that found in the literature [51,52]. For this reason, an inoculum containing 1 x 10^8^ CFU·mL^−1^ of *A. naeslundii* or 1 × 10^4^ CFU·mL^−1^ of *S. mutans* was used.

The fact that *A. naeslundii* maintained viability in either single- or dual-species biofilms, together with the fact that *S. mutans* viability reduced by 10.11% in dual-species biofilms, indicates that *A. naeslundii* may have inhibited *S. mutans* growth. On the other hand, it is noteworthy that the higher cell concentration in dual-species biofilms might have caused a nutrient competition between the species. These observations give important insights into the role of *A. naeslundii* in initial biofilm growth and in the biofilm ecological balance, as previously hypothesized by Takahashi and Nyvad [9]. The mechanisms by which *A. naeslundii* influences *S. mutans*—whether by growth inhibition or by overcoming nutritional limitations—should be addressed in the future.

The ecological balance was also maintained by the control of biofilm acidification, which was shown by the reduction in lactic acid production by the dual-species biofilms. The latter finding may be explained by the ability of *A. naeslundii* to convert lactate into weaker acids [33] to produce nitrite that inhibits bacterial acid production [53] and urea that neutralizes acids [31]. It is important to note that the present study evaluated the interaction of *S. mutans* and *A. naeslundii* at early stages of biofilm growth. The interaction between these two bacterial species at later stages of biofilm development is worth studying, since the cariogenicity of *A. naeslundii* may be enhanced if frequent acid challenges occur in biofilms [9]. 

The hypothesis that lactic acid produced by *S. mutans* may be either neutralized or further metabolized by *A. naeslundii* was corroborated by the fact that, in dual-species biofilms, *S. mutans* viability was reduced by 10.11% but lactic acid production was reduced by 50%. Thus, the reduction in lactic acid production was not proportional to the reduction in *S. mutans* viability. This finding is also supported by the absence of statistical differences between single- and dual-species biofilms in overall metabolic activity, as observed by the XTT reduction assay. Thus, we hypothesize that *A. naeslundii* is able to impair *S. mutans* growth. More important, however, is the ability of *A. naeslundii* to control the acidogenicity of biofilms. 

The production of the extracellular matrix is of great importance for the establishment of dental biofilms [54]. Proteins are an important component of biofilms, since they not only form the extracellular matrix but also stabilize it [4] and may degrade during starvation periods [55]. Biomass evaluation provides important information about the overall composition of biofilm, including cells (viable or non-viable) and extracellular components (polysaccharides, proteins, DNA) [56,57,58]. In the present study, there were no differences in total protein concentration in the biofilm matrix and in biomass between the different growth conditions (Figure 2), which suggests that the interaction of the studied species does not affect biofilm matrix protein concentration. 

Antimicrobial efficacy tests should preferably be performed using laboratory methods similar to the environment in which the biofilm is usually found. However, most studies of antimicrobial substances have been performed using static systems, which are not able to simulate shear forces created by salivary flow [59]. The hydrodynamic stress provided by flow systems allows biofilm formation with similar in vivo architecture and antimicrobial susceptibility [60,61]. The drip flow reactor (DFR) was developed and validated by the Standardized Biofilm Methods Laboratory of the Centre for Biofilm Engineering for growing, treating, sampling and analysing *Pseudomonas aeruginosa* biofilms [62]. This reactor is a good choice to model an oral biofilm because it offers a continuous low fluid shear that simulates salivary flow and clearance [63]. Moreover, it is easy to handle and can be purchased at a low cost. There are some studies that grew *Streptococcus mutans* biofilms in DFR to evaluate the antibacterial effect of restorative materials or substances [63,64,65,66]. However, to date, this reactor has not been used to study the interaction between cariogenic species. The DFR was chosen because it allows the creation of shear forces that mimic oral cavity conditions. 

With the DFR, statistically significant differences between treated (chlorhexidine) and control (NaCl-treated) biofilms were observed with approximately 1 log reduction in the treated biofilms (Figure 3). These results are closer to those observed in situ [67] and in vivo [68] trials. Thus, the results showed that the chosen experimental design was suitable for growing dental biofilms and testing the chlorhexidine susceptibility of single- and dual-species biofilms. Treatment time was increased to 2 min instead of using the standard clinical protocol for chlorhexidine mouthwash (30–60 s) because oral surfaces, dental pellicle and saliva are absent in the DFR. These structures retain chlorhexidine for an extended period, ensuring chlorhexidine substantivity [27]. Moreover, the present study comprehensively showed the antimicrobial activity of chlorhexidine against biofilms formed and tested under conditions closer to those found in the oral cavity [66]. This simplified flux reactor model also allowed biofilm growth in the absence of a salivary pellicle, which is in agreement with previous studies [69,70]. To the best of our knowledge, this is the first time that DFR has been used to study the stress response to chlorhexidine of oral bacteria.

An important finding of this study was that the presence of *A. naeslundii* increased *S. mutans’* chlorhexidine resistance in dual-species biofilms (Figure 3). Several factors may account for this finding. *Actinomyces* spp. are early colonizers that coaggregate with *S. mutans* [71]. This coaggregation was apparent in CLSM as clusters (Figure 4). This spatial arrangement may have acted as a physical barrier and might have affected chlorhexidine diffusion into the biofilm [72]. Furthermore, changes in bacterial cariogenicity and gene expression may have occurred [14]. The molecular mechanisms involved in this increased resistance were not studied in this investigation, but are an area of active investigation. Further studies using biofilms at different ages should be carried out to evaluate if this difference persists as biofilms age. Our hypotheses for the apparent conflict between biomass results and CLSM are: (a) *S. mutans* and *A. naeslundii* coagregate (Al-Ahmad et al., 2007; Kneist et al., 2012), which might have promoted the formation of microcolonies, as observed in CLSM images and as previously demonstrated (Bowen et al., 2011); (b) previous works have showed a lack of correlation between biochemical analysis of biofilms and CLSM results [29]. As previously suggested, the presence of another microorganism may influence biofilm architecture, as well as contribute to its survival and resistance to antimicrobials [25,26,27,28,73]. In the present study, the presence of larger clusters was observed in *S. mutans* and *A. naeslundii* dual-species biofilms. This finding might be related to a decrease in the chlorhexidine penetration in biofilm, which should be further evaluated.

While much effort has been devoted to understanding the molecular mechanisms of adherence, biofilm development and the expression of cariogenicity genes by *S. mutans* in pure cultures, there are still large gaps in our knowledge of how these molecular mechanisms translate to the phenotypic characteristics of this species in mixed communities. The outcomes of the present study raise questions about the mechanisms involved in *S. mutans* and *A. naeslundii* interaction and how this interaction may influence the occurrence of dental caries. The improvement of this understanding is the first step to better understanding the role of each species in caries pathogenesis and moving towards alternative therapies for the control and maintenance of oral health. 

## Supplementary Materials

The following are available online at https://www.mdpi.com/2076-2607/8/2/194/s1, Supplementary Material 1: Establishment of differential counting and dual-species biofilm growth, Supplementary Material 2: DFR validation. Supplementary Material 3: ANOVA summary.
