# Supplementary material for: Streptococcus mutans and Actinomyces naeslundii Interaction in Dual-Species Biofilm
Source: Microorganisms. 2020 Jan 31;8(2):194. doi: 10.3390/microorganisms8020194 (PMC7074783; doi:10.3390/microorganisms8020194)
Supplement: Supplementary file 1 [file microorganisms-08-00194-s001.pdf]

microorganisms

Supplementary Material 1: Establishment of differential counting and dual-species biofilm growth

As a methodology for the differential counting of *S. mutans* and *A. naeslundii* was not found in the literature, the authors carried out a validation study. Frozen stocks were streaked separately in agar, as previously described. Next, bacterial suspensions containing 10^8 CFU mL^{-1} of each species were prepared in 0.9% NaCl. Bacterial suspensions were streaked in BHI blood agar either individually or in combination at a 1:1 ratio (*S. mutans* + *A. naeslundii*). The plates were incubated at 5% de CO_2 and 37 °C for 72 h. Next, the colony morphology of individual suspensions was compared to that of combined suspension using a stereomicroscope (077148, Quimis, Diadema, SP, Brazil, 20x magnification).

Pilot studies were performed to find the appropriate concentration for growing *S. mutans* and *A. naeslundii* in dual-species biofilms. Frozen stocks were streaked separately in agar and incubated as described above. Next, agar cultures were used to obtain bacterial suspension in sterilized 0.9% NaCl containing 1×10^8 CFU mL^{-1} (*A. naeslundii*) or 10^4–10^7 CFU mL^{-1} (*S. mutans*).

For the growth of single-species biofilms, 3 mL of each bacterial suspension was individually added to 27 mL of BHI broth supplemented with 0.2% (v/v) (BHI-S broth). For the growth of dual-species biofilms, 3 mL of *S. mutans* suspension was combined with 3 mL of *A. naeslundii* suspension in 24 mL of BHI-S broth. Biofilms grew on the surface of hydroxyapatite discs (n = 6) in BHI-S broth (1.5 mL) for 24 h at 37 °C / 5% CO_2.

Hydroxyapatite discs (n = 6/group) were placed in a vertical position without touching the wall of 24-well plates, according to the model proposed by Exterkate et al. (2010), modified by Albuquerque et al. (2018). Next, the discs were immersed in 24-well plates containing 1.5 mL of the inocula and the plates were incubated in 5% CO_2 at 37 °C. The experiment was repeated twice.

For viable cell quantification, the discs were washed by immersion ten times in 0.9% NaCl and biofilms were re-suspended in 2 mL 0.9% NaCl by sonication (10 s, 42 kHz) (Albuquerque et al. 2018). Dual-species biofilm suspensions were plated in triplicate in BHI blood agar. The plates were incubated in 5% CO_2 at 37 °C for 72 h. Differential counting was carried out as previously standardized. Bacterial viability was expressed as the mean ± standard deviation log CFU mL^{-1}.

Results

S. mutans colonies were whitish, circular, with undulated margins, slightly umbonated, small (Ø 2 mm), hard and adherent to the culture medium. *A. naeslundii* colonies were circular, small (Ø 2 mm), opaque, with regular margins, convex and milky-white (Figure S1).

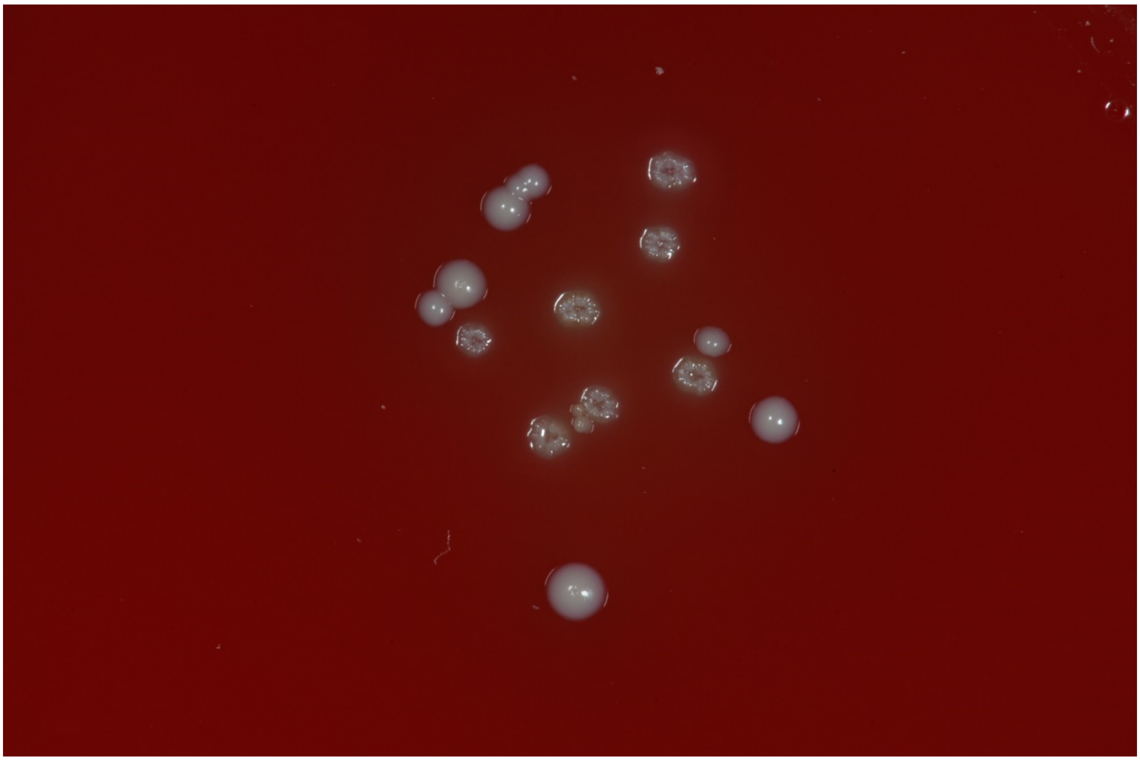

Figure S1. Differential counting of *S. mutans* (*) and *A. naeslundii* (**).

Similar concentrations of *S. mutans* and *A. naeslundii* were found only if *S. mutans'* initial inoculum concentration was 10^4 CFU mL^{-1}. This concentration was chosen for further evaluations (Figure S2).

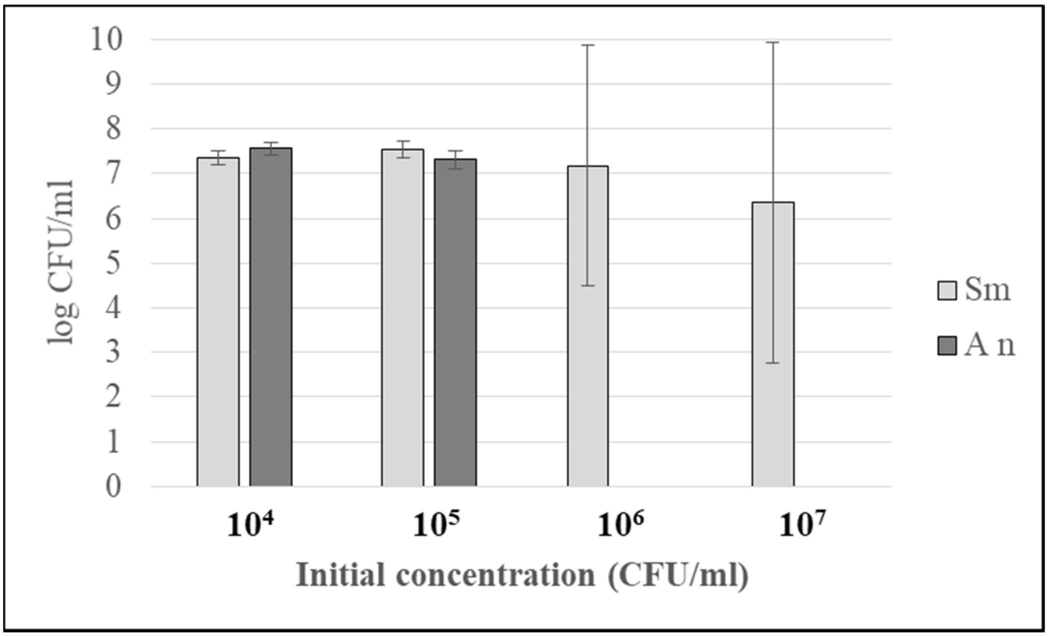

Figure S2. Viable cells (log CFU/mL) in dual-species biofilms according to the initial bacterial concentration of *S. mutans* suspension (10^4, 10^5, 10^6 and 10^7 CFU/mL). *A. naeslundii* concentration was 10^8 CFU/mL. "Sm": *Streptococcus mutans*; An: *Actinomyces naeslundii*.

Supplementary Material 2: DFR validation

The validation of DFR to form biofilms using oral microorganisms was initially performed using *S. mutans* mono-species biofilms. DFR channels were inoculated with 1.0 ml of cell suspensions at 10^2

CFU/ml or 10^4 CFU/ml and incubated for 1 h at 37 °C / 5% CO_2 with the DFRs in a horizontal position. The medium consisted of full-strength BHI broth supplemented with 0.5% sucrose. For each bacterial concentration, five independent experiments were carried out.

After 24 h, each channel was rinsed with 10 ml of 0.9% NaCl to remove residual growth medium and planktonic cells. DFR was placed in a horizontal position and biofilms were treated for 2 min with 20 ml of 0.9% NaCl (control) or 0.2% chlorhexidine solution, freshly prepared on the day of use. After treatments, 4 h of saline solution (NaCl 0.9%) flow at 10 ml/h rate was used as a wash-out step, in order to simulate salivary clearance.

Next, the slides were removed from the DFR and scraped in 10 ml of 0.9% NaCl. Slides and cell suspensions were vortexed for 30 s, sonicated for 2 min, and vortexed for an additional 30 s to remove and suspend biofilm cells. Cell suspensions were serially diluted and plated on BHI agar. Plates were incubated for 48 h at 37 °C / 5% CO_2. Colony-forming units were counted and the results were expressed as log CFU/mL. Log reduction was calculated as the difference between the log CFU/mL of the control group (0.9% NaCl) and chlorhexidine group.

Results

Table S1 shows that the *S. mutans* concentration of mono-species biofilms increased with the increasing concentration of initial inoculum. However, no statistically significant differences were observed in log reduction between the different inoculum concentrations used. This indicates that chlorhexidine treatment affected the biofilms similarly, in spite of the concentration of *S. mutans* used to start the biofilm formation.

Table S1. *Streptococcus mutans* cell counts (mean ± standard deviation) detected in 24 h biofilms according to initial inoculum suspension concentration (10^2 or 10^4 CFU/mL).

initial inoculum concentration (CFU/ml)	log CFU/mL*	log reduction†
10^2	6.80 ± 0.40[a]	2.36 ± 0.8[a]
10^4	8.22 ± 0.51[b]	1.58 ± 0.89[a]

*log CFU/mL: *S. mutans* concentration in 24 h biofilms after treatment with 0.9% NaCl.

† log reduction: difference between log CFU/mL of the control group (0.9% NaCl) and chlorhexidine group.

Means followed by different lowercase letters indicate statistically significant differences between biofilms within each column (unpaired t test $p < 0.05$).

Supplementary Material 3—ANOVA summary

Table S1. Stress response of single- and dual-species biofilms using DFR. Summary of two-way ANOVA for bacterial viability in single- and dual-species biofilms of *S. mutans* and *A. naeslundii* after treatment with chlorhexidine. The variables analysed were: treatment solution (saline or chlorhexidine) and biofilm type (single- or dual-species biofilms).

Source	df	SS	MS	F	p
Treatment solution	1	39.370	39.370	105.032	<0.001*
Culture condition	3	1.574	0.525	1.400	0.261
Treatment *vs* culture	3	7.704	2.568	6.851	0.001*

df = degrees of freedom; MS = mean square; F = MS factor/ MS residual; p = probability of significance, α=0.050. *statistically significant differences.
